# Comparison of prognosis between coronary computed tomography angiography versus invasive coronary angiography for stable coronary artery disease: a systematic review and meta-analysis

**DOI:** 10.3389/fcvm.2023.1010536

**Published:** 2023-05-05

**Authors:** Qingya Xie, Lingling Zhou, Ying Li, Ruizhe Zhang, Han Wei, Gaoxiang Ma, Yuping Tang, Pingxi Xiao

**Affiliations:** ^1^ Department of Cardiology, Sir Run Run Hospital, Nanjing Medical University, Nanjing, China; ^2^ Department of Orthopaedic Surgery, Children’s Hospital of Nanjing Medical University, Nanjing, China; ^3^ State Key Laboratory of Natural Medicines, School of Traditional Chinese Pharmacy, China Pharmaceutical University, Nanjing, China; ^4^ Department of Cardiology, Nanjing Drum Tower Hospital Group Suqian Hospital, Suqian, China; ^5^ Department of Cardiology, The Forth Affiliated Hospital, Nanjing Medical University, Nanjing, China

**Keywords:** coronary computed tomography angiography, invasive coronary angiography, stable coronary artery disease, meta-analysis, review

## Abstract

**Background:**

The impact of using invasive coronary angiography (ICA) or coronary computed tomography angiography (CCTA) as an initial examination on the incidence of major adverse cardiovascular events (MACEs) in patients with stable coronary artery disease and the occurrence of major operation-related complications is uncertain.

**Objective:**

This study aimed to explore the effects of ICA vs. CCTA on MACEs, all-cause death, and major operation-related complications.

**Methods:**

A systematic search of electronic databases (PubMed and Embase) was conducted for randomized controlled trials and observational studies comparing MACEs between ICA and CCTA from January 2012 to May 2022. The primary outcome measure was analyzed using a random-effects model as a pooled odds ratio (OR). The main observations were MACEs, all-cause death, and major operation-related complications.

**Results:**

A total of six studies, comprising 26,548 patients, met the inclusion criteria (ICA *n* = 8,472; CCTA *n* = 18,076). There were statistically significant differences between ICA and CCTA for MACE [OR 1.37; 95% confidence interval (CI), 1.06–1.77; *p* = 0.02], all-cause death (OR 1.56; 95% CI, 1.38–1.78; *p* < 0.00001), and major operation-related complications (OR 2.10; 95% CI, 1.23–3.61; *p* = 0.007) among patients with stable coronary artery disease. Subgroup analysis demonstrated statistically significant results in the impact of ICA or CCTA on MACEs according to the length of follow-up. Compared to CCTA, ICA was related to a higher incidence of MACEs in the subgroup with a short follow-up (≤3 years) (OR 1.74; 95% CI, 1.54–1.96; *p* < 0.00001).

**Conclusions:**

Among patients with stable coronary artery disease, an initial examination with ICA was significantly associated with the risk of MACEs, all-cause death, and major procedure-related complications compared to CCTA in this meta-analysis.

## Introduction

Stable coronary artery disease (SCAD) is generally characterized by episodes of reversible myocardial demand/supply mismatch related to ischemia or hypoxia. SCAD is usually inducible by exercise, emotion, or other stress and is reproducible, but it may also occur spontaneously. At present, SCAD has a high incidence and degree of risk. As SCAD is so multifaceted, its prevalence and incidence have been difficult to assess; these figures vary greatly among studies depending on the definition used. According to the American Society of Cardiovascular Disease (ACC) in 2016, the incidence of stable coronary heart disease is twice as high as that of myocardial infarction, and is expected to be as high as 18% of the adult population in 2030 (1). Currently, the main diagnosis of SCAD includes clinical evaluation, noninvasive tests such as stress tests or coronary computed tomography angiography (CCTA), and invasive coronary angiography (ICA) (2).

It is well known that ICA is the reference standard for the diagnosis of stable angina. CCTA has attracted attention because it is a noninvasive alternative that can rule out obstructive coronary artery disease (CAD) with a low risk of adverse events. However, higher referral rates for ICA and vascular reconstruction were both highlighted as potential shortcomings of the CCTA priority strategy (3). A study showed that the correlation coefficient between ICA and CCTA for the diagnosis of patients with suspected CAD was 0.9 (4). A meta-analysis revealed that the sensitivity of CCTA for the diagnosis of coronary artery patients using ICA as the diagnostic criterion was 88% (5).

A study has demonstrated that the combined rate of major operation-related complications associated with ICA diagnostic procedures is in the range of 0.5%–2% (6). Although the incidence of major operation-related complications related to ICA diagnostic procedures is low, adverse events are increasingly recognized as affecting patients' medical compliance. In conclusion, previous meta-analyses have evaluated the diagnostic performance of noninvasive tests compared with ICA (5, 7, 8). However, ICA has not been systematically evaluated in comparison with CCTA in predicting the occurrence of major adverse cardiovascular events (MACEs) and major operation-related complications. Therefore, we conducted a meta-analysis to systematically investigate the advantages and disadvantages of ICA and CCTA in their prediction of MACEs and major operation-related complications to guide the diagnosis and treatment of patients with SCAD.

## Methods

### Search strategy

We used the PubMed and EMBASE databases to conduct a literature search of relevant clinical studies from January 2012 to May 2022. The literature search was limited to human clinical studies, and the search terms were “coronary artery disease”, “ICA” and “CCTA”. The full search terms are illustrated in Supplementary Appendix S1, S2. We reviewed each publication and included only the latest or most complete clinical trial reports when duplicates were found.

### Inclusion and exclusion criteria

Studies that met the predefined criteria were included in this review: (1) ICA for one group and CCTA for another group; (2) inclusion of patients with stable known or suspected CAD ((i) with stable angina or other symptoms related to CAD, such as dyspnea; (ii) patients with previously known nonobstructive CAD symptoms who were asymptomatic after treatment and required regular follow-ups; (iii) those who first reported symptoms and were judged to be in a chronic stable state) (2); and (3) data on MACE and major operation-related complications were included, as well as the sample size available for analysis. Studies were considered ineligible when one of the following occurred: (1) the study did not include patients with ICA and/or CCTA and/or SCAD; (2) study methods or results were not available from the article or investigator. The selection of relevant literature was independently conducted by two researchers, and disagreements were resolved by consulting a third reviewer.

### Data extraction and quality assessment

Two reviewers independently extracted the data from the selected studies using a standardized data extraction form, with disagreements resolved via consensus or by third reviewers when necessary. For each study, the following information was extracted: name of first author and year of publication, study characteristics, patient characteristics, number of patients, median follow-up time, and various outcomes. MACEs and major operation-related complications were extracted from the safety data of each trial. MACE included death, myocardial infarction, late revascularization, cardiac arrhythmia or chest pain requiring hospitalization, cerebrovascular events, hospital admission for refractory myocardial ischemia or congestive heart failure, with death and myocardial infarction being common among studies. Major operation-related complications included nonfatal myocardial infarction, nonfatal stroke, cardiac arrhythmia (ventricular tachycardia or fibrillation), complications prolonging hospitalization by ≥24 h, dissection of the coronary artery or aorta, cardiac arrest, cardiac tamponade, local vascular perforation, and severe allergic reactions.

The quality evaluation was assessed by two researchers independently. The JADAD Scale was used to evaluate randomized controlled trials, and the Newcastle‒Ottawa Scale (NOS) was used for cohort trials (9, 10). The JADAD Scale comprised 4 items, with a scale ranging from 0 to 7 (Supplementary Table S1). Scores of 4–7 were regarded as high quality, and scores of 1–3 were regarded as low quality. For the NOS, an overall quality score contains 8 items rated on a scale of 0–9 stars (Supplementary Table S2). When a study obtained more than 6 scores, it was regarded as high quality. When a study obtained scores of 4–6, it was regarded as moderate quality. According to the JADAD and NOS, all studies involved were of high quality (Supplementary Table S3).

### Statistical analysis

For each meta-analysis, *I*^2^ statistics were first calculated to assess the heterogeneity among the proportions of the included trials. Values of 25%, 50%, and 75% were regarded as low, moderate, and high heterogeneity, respectively, on the basis of the *I*^2^ statistic. Considering heterogeneity, data were analyzed using a random-effects model (11). The combined effect estimates are shown as pooled odds ratios with 95% confidence intervals (CIs) and *p* values. Sensitivity analysis was performed by omitting one study at a time. Publication bias was assessed using funnel plots and Begg's test. We also carried out subgroup analyses according to the type of study and the length of follow-up. Statistical analysis was performed using the Cochrane Review Manager (RevMan, version 5.4; The Cochrane Community, London, UK) and STATA version 15 (College State, TX).

## Results

### Study selection and characteristics

**Figure 1:**
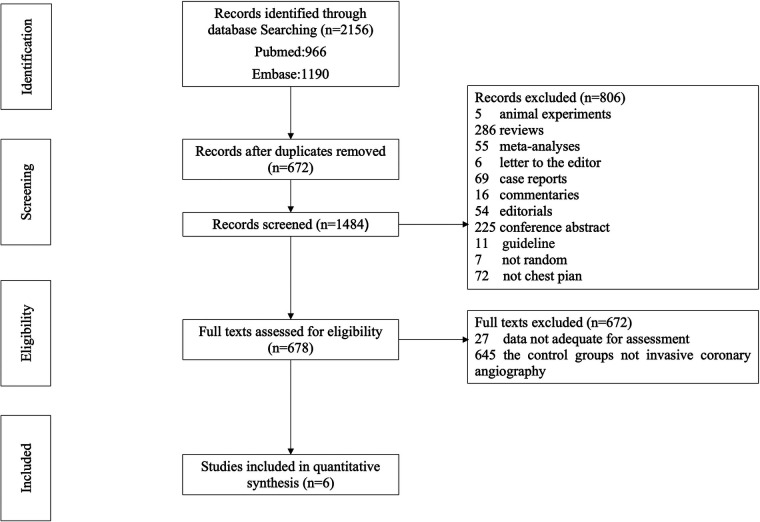
Flowchart for process of study selection.

The search strategy is shown in Figure 1. Our search yielded 2,156 clinical studies relevant to SCAD: 966 articles from PubMed and 1,190 articles from Embase. Following deduplication, 1,484 titles were screened. After evaluating the title and abstract of each study, 806 studies were initially excluded. Another 672 trials were excluded after reviewing the full text, as they failed to fulfill the inclusion criteria. Finally, we included 6 clinical trials for the purpose of analysis (12–17). Data from 26,548 patients were available for the meta-analysis. The baseline characteristics of patients and studies are listed in Table 1. The demographics, comorbidities, and study characteristics of the studies are listed in Table 2. The follow-up period ranged from in-hospital to a maximum of 7 years. The average age was 60.2 and 60.3 years in the ICA and CCTA groups, respectively. Totals of 57.9% and 50.0% of patients were male in the ICA and CCTA groups, respectively. The incidences of hypertension in each group were 62.3% and 53.9% for the ICA and CCTA groups, respectively. The incidences of diabetes mellitus in each group were 14.2% and 10.2% for the ICA and CCTA arms, respectively. The incidences of dyslipidemia in each group were 51.1% and 42.2% for the ICA and CCTA arms, respectively. The incidences of current smokers in each group were 34.1% and 33.5% for the ICA and CCTA arms, respectively.

**Table 1:** Characteristics of studies included in the meta-analysis.

Author	Design	Year	Primary endpoint	Treatment regimens per arm	Number of Enrolled patients, n	Duration of follow-up, year
Dewey	Observational trail	2021	Major adverse cardiovascular event	Arm A CTA-CTP	147	5
				Arm B ICA-SPECT	235	
Maurovich	RCT	2022	Major adverse cardiovascular event	Arm A CTA	1,808	3.5
				Arm B ICA	1,753	
Kofoed	RCT	2021	Death from any cause, non-fatal recurrent myocardial infarction, refractory myocardial ischemia, clinical heart failure	Arm A CTA	260	4.2
				Arm B ICA	324	
Shen	RCT	2020	Major adverse cardiovascular event	Arm A ICA	51	1
				Arm B CTA	51	
Winther	Observational trial	2022	All-cause death and myocardial infarction	Arm A CTA	25,026	3.9
				Arm B CTA	15,643	3.4
				Arm C CTA-MPI	3,547	3.2
				Arm D CTA-ICA	9,135	4.1
Dewey	RCT	2016	Major procedural complications	Arm A CTA	167	7
				Arm B ICA	162	

RCT, randomized control trial; CTA, computed tomography angiography; ICA, invasive coronary angiography; SPECT, single-photon emission computed tomography; CTP, computed tomography myocardial perfusion imaging; MPI, myocardial perfusion imaging.

**Table 2:** Baseline raw data demographics, comorbidities, and study characteristics of studies included in the meta-analysis.

Study	Dewey 2021	Maurovich 2022	Kofoed 2021	Shen 2020	Winther 2022	Dewey 2016
Sample (*n*) CTA/ICA	147/235	1,808/1,753	260/324	51/51	15,643/9,135	167/162
Age	NA	61.3/60.6	58.9/60.1	60/57	61.1/62.9	60.4/60.4
Male (%)	NA	1,019 (56.4)/983 (56.1)	113 (43.5)/149 (46.0)	31 (60.8)/30 (58.8)	7,714 (49.3)/5,371 (58.8)	79 (47.3)/84 (51.9)
**CAD risk factors**						
Hypertension	NA	1,102/1,020	105/137	24/25	8,317/5,821	111/112
Diabetes mellitus	NA	263/294	NA	9/9	1,509/1,243	15/30
Dyslipidemia	NA	874/832	NA	17/19	6,467/4,737	95/81
Current smoker	NA	343/300	60/82	20/16	5,539/3,469	41/34
Prior MI	NA	NA	20/37	NA	NA	NA
Prior PCI	NA	195/253	12/28	NA	NA	NA
Prior CABG	NA	39/62	NA	NA	NA	NA
**Clinical presentation**						
Typical angina	NA	232/275	NA	NA	1,202/1,902	65/79
Atypical angina	NA	843/805	NA	NA	6,169/3,473	NA
Nonanginal chest pain	NA	677/634	NA	NA	4,203/1,440	97/80
Other	NA	56/39	NA	NA	4,069/2,316	5/3

NA, not available.

### Pooled results of the meta-analysis

**Figure 2:**
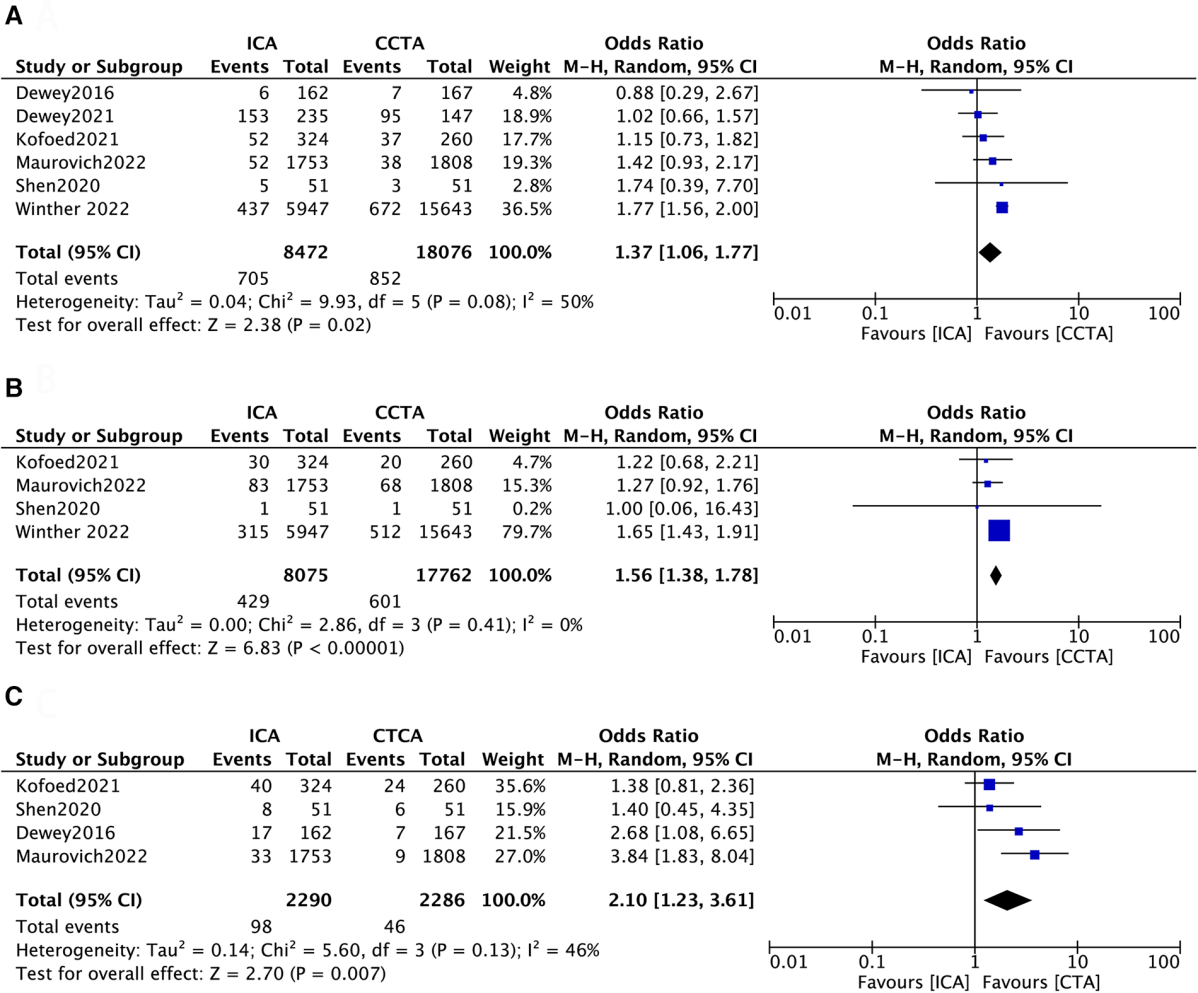
Forest plots of (**A**) major adverse cardiac events, (**B**) all-cause death and (**C**) operation-related complications.

All included studies reported data on MACEs (12–17). Four of the six studies assessed all-cause death during follow-up (13–16). Four studies reported major operation-related complications (13–17). There were statistically significant results between the two groups in terms of MACE (ICA = 705[8.3%] vs. CCTA = 852[4.7%]; OR 1.37; 95% CI, 1.06–1.77; *p* = 0.02, Figure 2A) and all-cause death (ICA = 429[5.3%] vs. CCTA = 601[3.4%]; OR 1.56; 95% CI, 1.38–1.78; *p *< 0.00001, Figure 2B). The overall OR of major operation-related complications with ICA was 2.10-fold higher (95% CI, 1.23–3.61; *p* = 0.007, Figure 2C) than that in patients with CCTA.

### Heterogeneity and publication bias

Given the moderate heterogeneity of MACE and the low heterogeneity of major operation-related complications shown in the forest plots, the sensitivity analysis was conducted by omitting one study at a time (Supplementary Tables S4, S5). In terms of MACEs, no significant difference in the effect of initial examination using ICA and CCTA was observed after removing the study of Winther 2022 (OR 1.19; 95% CI, 0.93–1.51; *p *= 0.17), with heterogeneity decreasing from 50% to 0% (Supplementary Table S4). It can also be seen that the results were affected in the same way after deleting the study of Maurovich 2022 (OR 1.32; 95% CI, 0.94–1.85; *p *= 0.10, Supplementary Table S4). In terms of major operation-related complications, after deleting the study of Dewey 2016, no statistically significant effect of initial examination using ICA and CCTA was observed (OR 1.98; 95% CI, 0.97–4.02; *p *= 0.06, Supplementary Table S5). Otherwise, the results were not overly influenced by other single studies. To evaluate publication bias, funnel plots were constructed (Supplementary Figure S1). The results of Begg's test regarding MACEs (*p *= 0.38) and major operation-related complications (*p *= 0.68) suggested no significant publication bias.

### Subgroup analysis

**Figure 3:**
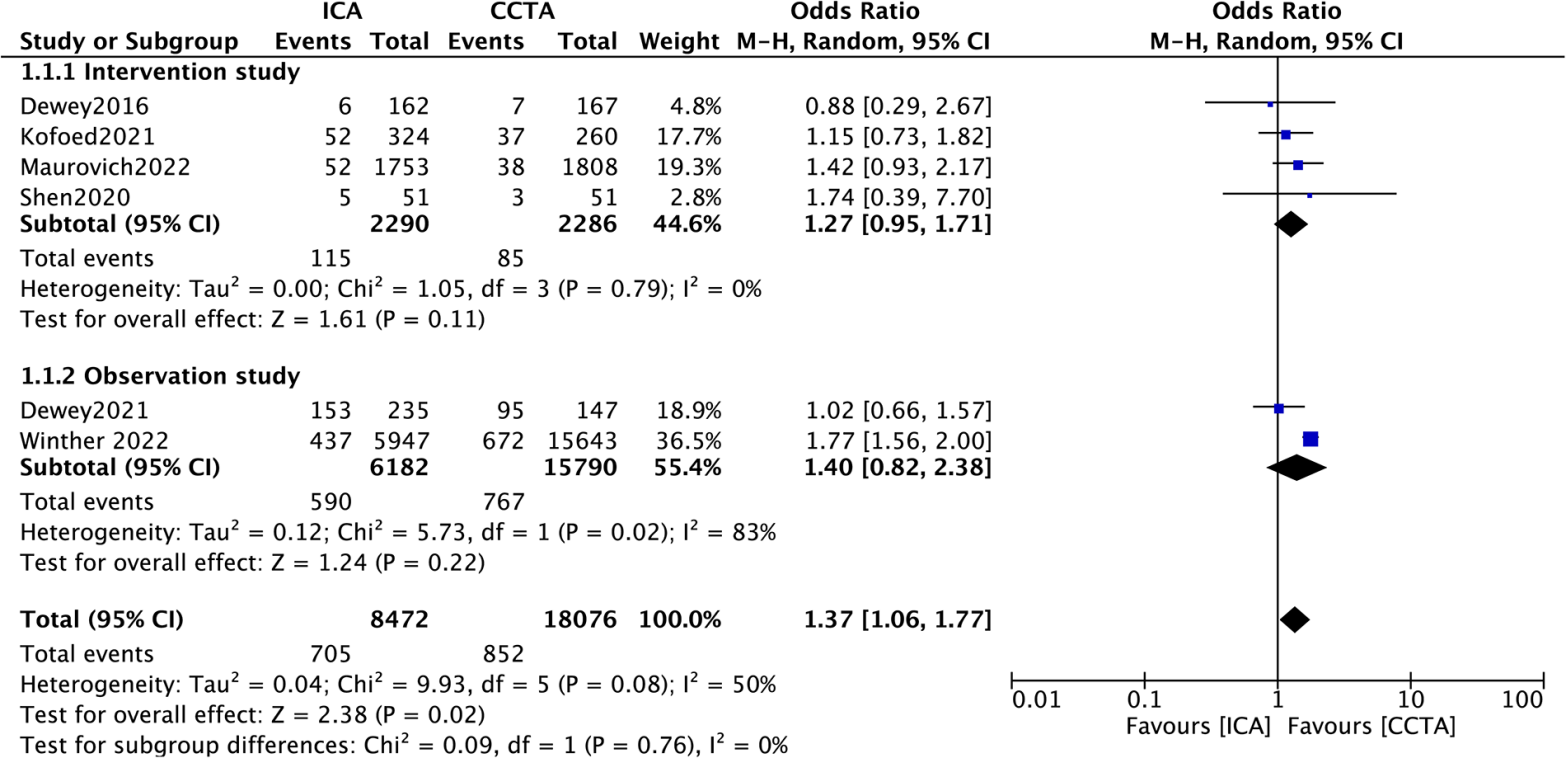
Subgroup analysis of MACE according to the type of study.

**Figure 4:**
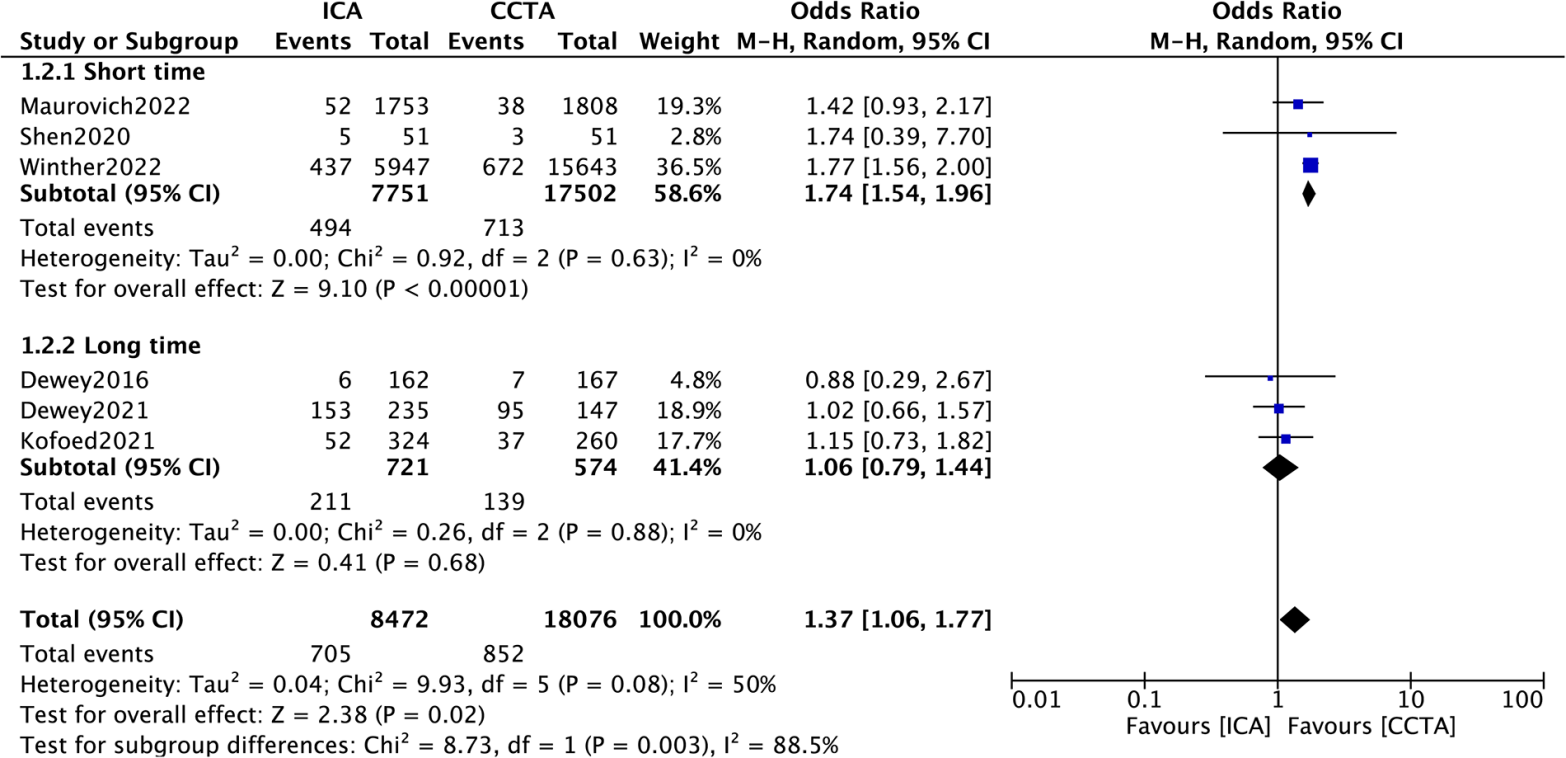
Subgroup analysis of MACE according to the study length of follow-up.

In consideration of the heterogeneity for the MACE outcome, we performed subgroup analysis to identify the potential source of heterogeneity. Subgroup analysis based on study type (intervention/observation)showed no statistically significant result in the effect of ICA or CCTA on MACE between the subgroups (Figure 3, *p* = 0.76). Specifically, the pooled ORs were 1.27 (95% CI, 0.95–1.71; *p *= 0.11) in the interventional study subgroup and 1.40 (95% CI, 0.82–2.38*; p *= 0.22) in the observational study subgroup. In addition, subgroup analysis for length of follow-up showed a statistically significant result between the subgroups (Figure 4, *p* = 0.003). The follow-up period varied from in-hospital to a minimum of one year to a maximum of seven years. Thus, a subgroup analysis was performed with a follow-up period limited to three years. The pooled ORs were 1.74 (95% CI, 1.54–1.96; *p < *0.00001) for the short follow-up subgroup (≤3 years) and 1.06 (95% CI, 0.79–1.44*; p *= 0.68) for the long follow-up subgroup (>3 years).

## Discussion

The objective of our study was to assess the potential differences in the impact of the initial examination using ICA or CCTA on major adverse cardiovascular events (MACEs), all-cause death, and major operation-related complications. This meta-analysis analyzed data from six studies comprising 26,548 patients. The key findings of this study are as follows: (1) There were statistically significant differences between the two groups in terms of MACE, all-cause death, and major operation-related complications. The overall odds ratio (OR) of major operation-related complications with ICA was 2.10-fold higher. (2) This effect was observed for both the duration of follow-up (short-term) and type of study (observational study).

To our knowledge, previous meta-analyses have compared diagnostic rates between ICA and CCTA (5, 8, 18), but there is no relevant meta-analysis on the effect of ICA or CCTA on the risk of MACE and the occurrence of major operation-related complications. Despite the technical differences between ICA and CCTA, previous studies have shown no significant differences in diagnostic outcomes between the two diagnostic modalities of ICA and CCTA among patients with stable coronary arteries (19, 20). Therefore, it is particularly important to study the impact of the initial examination using ICA and CCTA on the risk of MACEs and the impact on major operation-related complications.

CCTA requires only an intravenous injection of contrast and computed tomography of the area to be imaged (21, 22). Compared to ICA, it can better show the opening of the vessels and can well determine the nature of the coronary plaque. Additionally, CCTA can show lesions outside the coronary arteries, such as tumors. Finally, CCTA is noninvasive, less risky, has fewer side effects, and has higher patient compliance. However, CCTA requires heart rate control and has a high sensitivity and low specificity for calcified lesions. ICA inserts a catheter into the coronary artery after puncturing through the femoral or radial artery and selectively injects contrast into the coronary artery. Compared to CCTA, ICA is intuitive and accurate, and the results are reliable (23). In addition, ICA allows the direct selection of treatment options. Additionally, there is no need to control the heart rate or diet before the procedure. However, ICA is an invasive test that is more invasive and may cause some complications. Results of two large trials, the PROMISE (24) and SCOT-HEART trials (25), which compare CT with functional testing in patients with SCAD. In both of those trials, investigators found that CT was as good as or better than functional test-ing as a preliminary evaluation before possible ICA. Our meta-analysis confirmed the safety of a CT strategy and showed results that were similar to those with ICA. Improvement in quality of life are key objectives in the treatment of patients with SCAD.The findings of this meta-analysis showed statistically significant results in the effect of initial examination using ICA and CCTA on MACE, but the incidence of major operation-related complications was lower in patients who used CCTA examination, an important outcome for the comparison of invasive and noninvasive management strategies. Therefore, in terms of accuracy, prediction of MACE, all-cause death and major operation-related complications, CCTA is more suitable for patients with SCAD.

Following the exclusion of Winther 2022, the differences between MACE and all-cause deaths disappeared, and the heterogeneity decreased from 50% to 0%. After a thorough evaluation, the literature was found to meet the inclusion criteria and was not excluded based on high heterogeneity. Subsequently, a subgroup analysis was conducted, and the findings suggested that the heterogeneity may be attributed to the type of study and length of follow-up. The use of ICA and CCTA for initial examination was statistically significant in subgroups with a short follow-up. Although the Winther2022 study was an observational study that is prone to bias when compared with randomized controlled trials, no obvious publication bias was observed in the results of the constructed funnel plot. In the future, to reduce the heterogeneity between studies and increase the accuracy of the analysis, studies with the same study type and follow-up time can be included for meta-analysis.

Several limitations of our study need to be acknowledged. First, the definition of MACEs varied among the included studies, which may have influenced the consistency of our findings. Second, SCAD incidence increases with age and is higher in middle-aged women than in men (26, 27). However, due to limited data, we were unable to perform subgroup analyses for different age groups or sexes. Therefore, we performed a pooled analysis of patients with SCAD in all age groups. Third, the severity of CAD among patients in the included studies varied to some extent, which may have impacted data analysis. Fourth, there was low to moderate heterogeneity among the studies, which may have influenced the reliability of our results. To address this issue, we used a random-effects model and performed subgroup analyses to explore possible sources of heterogeneity. The results showed that different follow-up times and study types might partly account for the heterogeneity. However, the results of the short follow-up time subgroup were statistically significant, while the results of the long follow-up time subgroup were not. This discrepancy needs to be further investigated by future studies. Additionally, the included studies did not have clear functional test data, which might have affected the reliability of our results. Finally, the sample size of our study was limited, which may have introduced bias. Moreover, due to the limited number of studies on this topic, the funnel plot was slightly asymmetric, and an accurate assessment of publication bias was not possible.

Given the limitations of the data, we were unable to perform subgroup analyses of patients with SACD in different age groups or subgroup analyses of sex. Therefore, we performed a pooled analysis of patients with SCAD in all age groups. Third, the severity of CAD patients in the included studies varied to a certain extent, which may have a certain impact on data analysis. Fourth, there was low to moderate heterogeneity among the meta-analysis studies, which may influence the reliability of the results. However, this is an inevitable problem. Therefore, we used a random effects model to complete the meta-analysis, conservatively accounting for heterogeneity. Additionally, subgroup analyses were performed to explore possible sources of heterogeneity, and the results showed that different follow-up times and study types might partly account for the heterogeneity. In addition, the results of the short follow-up time subgroup were statistically significant, while the results of the long follow-up time subgroup were not, which needs to be supplemented by more studies. Not all patients with stable coronary arteries end up having to undergo revascularization surgery, and the included studies did not have clear functional test data that might affect the reliability of the results. Finally, the effect of our study may be biased due to the size of the sample size. In addition, due to the limited number of studies on this topic, the funnel plot is slightly asymmetric. Therefore, an accurate assessment of publication bias is not possible.

## Conclusion

This meta-analysis compared the impact of initial examination with ICA or CCTA on the occurrence of MACEs and major operation-related complications among patients with SCAD. In terms of MACEs, all-cause death and major operation-related complications, there were all statistically significant results. In summary, CCTA is superior to ICA in reducing the incidence of MACEs, all-cause death, and major operation-related complications. Therefore, CCTA is a safer diagnostic method for patients with SCAD.

## Data Availability

The original contributions presented in the study are included in the article/**Supplementary Material**, further inquiries can be directed to the corresponding author/s.

## References

[1] Writing Group Members, Mozaffarian D Benjamin EJ Go AS Arnett DK Blaha MJ Cushman M Executive summary: heart disease and stroke statistics–2016 update: a report from the American heart association. Circulation. (2016) 133:447–54. 10.1161/CIR.0000000000000366 26811276

[2] Task Force Members A Montalescot G Sechtem U Achenbach S Andreotti F Arden C 2013 ESC guidelines on the management of stable coronary artery disease: the task force on the management of stable coronary artery disease of the European society of cardiology. Eur Heart J. (2013) 34:2949–3003. 10.1093/eurheartj/eht296 23996286

[3] Narula J Chandrashekhar Y Ahmadi A Abbara S Berman DS Blankstein R SCCT 2021 Expert consensus document on coronary computed tomographic angiography: a report of the society of cardiovascular computed tomography. J Cardiovasc Comput Tomogr. (2021) 15:192–217. 10.1016/j.jcct.2020.11.001 33303384 PMC8713482

[4] Takagi H Tanaka R Nagata K Ninomiya R Arakita K Schuijf JD Diagnostic performance of coronary CT angiography with ultra-high-resolution CT: comparison with invasive coronary angiography. Eur J Radiol. (2018) 101:30–7. 10.1016/j.ejrad.2018.01.030 29571798

[5] Pontone G Guaricci AI Palmer SC Andreini D Verdecchia M Fusini L Diagnostic performance of non-invasive imaging for stable coronary artery disease: a meta-analysis. Int J Cardiol. (2020) 300:276–81. 10.1016/j.ijcard.2019.10.046 31748186

[6] Arora N Matheny ME Sepke C Resnic FS. A propensity analysis of the risk of vascular complications after cardiac catheterization procedures with the use of vascular closure devices. Am Heart J. (2007) 153:606–11. 10.1016/j.ahj.2006.12.014 17383300

[7] Salavati A Radmanesh F Heidari K Dwamena BA Kelly AM Cronin P. Dual-source computed tomography angiography for diagnosis and assessment of coronary artery disease: systematic review and meta-analysis. J Cardiovasc Comput Tomogr. (2012) 6:78–90. 10.1016/j.jcct.2011.10.018 22226727

[8] Mowatt G Cook JA Hillis GS Walker S Fraser C Jia X 64-slice computed tomography angiography in the diagnosis and assessment of coronary artery disease: systematic review and meta-analysis. Heart. (2008) 94:1386–93. 10.1136/hrt.2008.145292 18669550

[9] Wells GA, Shea B, O’Connell D, Peterson J, Welch V, Losos M http://www.ohri.ca/programs/clinical_epidemiology/oxford.asp.

[10] Jadad AR Moore RA Carroll D Jenkinson C Reynolds DJ Gavaghan DJ Assessing the quality of reports of randomized clinical trials: is blinding necessary? Control Clin Trials. (1996) 17:1–12. 10.1016/0197-2456(95)00134-4 8721797

[11] Higgins JPT Thompson SG Deeks JJ Altman DG. Measuring inconsistency in meta-analyses. Br Med J. (2003) 327:557–60. 10.1136/bmj.327.7414.557 12958120 PMC192859

[12] Dewey M Rochitte CE Ostovaneh MR Chen MY George RT Niinuma H Prognostic value of noninvasive combined anatomic/functional assessment by cardiac CT in patients with suspected coronary artery disease - comparison with invasive coronary angiography and nuclear myocardial perfusion imaging for the five-year-follow up of the CORE320 multicenter study. J Cardiovasc Comput Tomogr. (2021) 15:485–91. 10.1016/j.jcct.2021.04.005 34024757 PMC8570979

[13] Kofoed KF Engstrøm T Sigvardsen PE Linde JJ Torp-Pedersen C de Knegt M Prognostic value of coronary CT angiography in patients with non-ST-segment elevation acute coronary syndromes. J Am Coll Cardiol. (2021) 77:1044–52. 10.1016/j.jacc.2020.12.037 33632478

[14] Maurovich-Horvat P Bosserdt M Kofoed KF Rieckmann N Benedek T Donnelly P CT or invasive coronary angiography in stable chest pain. N Engl J Med. (2022) 386:1591–602. 10.1056/NEJMoa2200963 35240010

[15] Shen SX Zhao ZL Du S Shi PF Ding SK Wang GG The role of coronary. CT angiography in improving the positive rate of coronary angiography in patients with low- to medium-risk non-ST-segment elevation myocardial infarction[M]. Chin Med J. (2020) 100:3255–60. 10.3760/cma.j.cn 112137-20200407-01096 10.3760/cma.j.cn112137-20200407-01096 33167114

[16] Winther S Andersen IT Gormsen LC Steffensen FH Nielsen LH Grove EL Prognostic value of myocardial perfusion imaging after first-line coronary computed tomography angiography: a multi-center cohort study. J Cardiovasc Comput Tomogr. (2022) 16:34–40. 10.1016/j.jcct.2021.08.001 34475016

[17] Dewey M Rief M Martus P Kendziora B Feger S Dreger H Evaluation of computed tomography in patients with atypical angina or chest pain clinically referred for invasive coronary angiography: randomised controlled trial. Br Med J. (2016) 355:i5441. 10.1136/bmj.i5441 27777234 PMC5076567

[18] Paech DC Weston AR. A systematic review of the clinical effectiveness of 64-slice or higher computed tomography angiography as an alternative to invasive coronary angiography in the investigation of suspected coronary artery disease. BMC Cardiovasc Disord. (2011) 11:32. 10.1186/1471-2261-11-32 21679468 PMC3141758

[19] Ehara M Surmely J-F Kawai M Katoh O Matsubara T Terashima M Diagnostic accuracy of 64-slice computed tomography for detecting angiographically significant coronary artery stenosis in an unselected consecutive patient population: comparison with conventional invasive angiography. Circ J. (2006) 70:564–71. 10.1253/circj.70.564 16636491

[20] Leber AW Knez A von Ziegler F Becker A Nikolaou K Paul S Quantification of obstructive and nonobstructive coronary lesions by 64-slice computed tomography: a comparative study with quantitative coronary angiography and intravascular ultrasound. J Am Coll Cardiol. (2005) 46:147–54. 10.1016/j.jacc.2005.03.071 15992649

[21] Miller JM Rochitte CE Dewey M Arbab-Zadeh A Niinuma H Gottlieb I Diagnostic performance of coronary angiography by 64-row CT. N Engl J Med. (2008) 359:2324–36. 10.1056/NEJMoa0806576 19038879

[22] Sato Y Matsumoto N Kato M Inoue F Horie T Kusama J Noninvasive assessment of coronary artery disease by multislice spiral computed tomography using a new retrospectively ECG-gated image reconstruction technique. Circ J. (2003) 67:401–5. 10.1253/circj.67.401 12736477

[23] Roffi M Patrono C Collet J-P Mueller C Valgimigli M Andreotti F 2015 ESC guidelines for the management of acute coronary syndromes in patients presenting without persistent ST-segment elevation: task force for the management of acute coronary syndromes in patients presenting without persistent ST-segment elevation of the European society of cardiology (ESC). Eur Heart J. (2016) 37:267–315. 10.1093/eurheartj/ehv320 26320110

[24] Douglas PS Hoffmann U Patel MR Mark DB Al-Khalidi HR Cavanaugh B Outcomes of anatomical versus functional testing for coronary artery dis- ease. N Engl J Med. (2015) 372:1291–300. 10.1056/NEJMoa1415516 25773919 PMC4473773

[25] Newby DE Adamson PD Berry C Boon BA Dweck MR Flather M Coronary CT angiography and 5-year risk of myocardial infarction. N Engl J Med. (2018) 379:924–33. 10.1056/NEJMoa1805971 30145934

[26] Reis SE Holubkov R Conrad Smith AJ Kelsey SF Sharaf BL Reichek N Coronary microvascular dysfunction is highly prevalent in women with chest pain in the absence of coronary artery disease: results from the NHLBI WISE study. Am Heart J. (2001) 141:735–41. 10.1067/mhj.2001.114198 11320360

[27] Han SH Bae JH Holmes DR Lennon RJ Eeckhout E Barsness GW Sex differences in atheroma burden and endothelial function in patients with early coronary atherosclerosis. Eur Heart J. (2008) 29:1359–69. 10.1093/eurheartj/ehn142 18424787

