# Supplementary material for: Comparison of prognosis between coronary computed tomography angiography versus invasive coronary angiography for stable coronary artery disease: a systematic review and meta-analysis
Source: Front Cardiovasc Med. 2023 May 5;10:1010536. doi: 10.3389/fcvm.2023.1010536 (PMC10196209; doi:10.3389/fcvm.2023.1010536)
Supplement: Supplementary file 1 [file Table1.docx]

***Supplementary Material***

**Content:
Supplementary Materials:
Supplementary Appendix 1:** Search terms for PubMed.
**Supplementary Appendix 2:** Search terms for Embase.
**Supplementary Table S1:** The Jadad Scale for assessing the quality for randomized controlled trials.
**Supplementary Table S2:** Newcastle-Ottawa Scale for assessing the quality for cohort trials.
**Supplementary Table S3:** The scores of quality assessment for randomized controlled trials and cohort trails.
**Supplementary Table S4:** Sensitivity analysis by detecting one study each time of Major adverse cardiac events.
**Supplementary Table S5:** Sensitivity analysis by detecting one study each time of Operation-related complications.
**Supplementary Figure S1:** Funnel plots for the assessment of publication bias in **(A)** Major adverse cardiac events, **(B)** Operation-related complications.

**Supplementary Appendix 1:** Search terms for PubMed.

 ((("Coronary Artery Disease"[Mesh]) OR (((((((((((((((Artery Disease, Coronary[Title/Abstract]) OR (Artery Diseases, Coronary[Title/Abstract])) OR (Coronary Artery Diseases[Title/Abstract])) OR (Left Main Coronary Artery Disease[Title/Abstract])) OR (Left Main Disease[Title/Abstract])) OR (Left Main Diseases[Title/Abstract])) OR (Left Main Coronary Disease[Title/Abstract])) OR (Coronary Arteriosclerosis[Title/Abstract])) OR (Arterioscleroses, Coronary[Title/Abstract])) OR (Coronary Arterioscleroses[Title/Abstract])) OR (Atherosclerosis, Coronary[Title/Abstract])) OR (Atheroscleroses, Coronary[Title/Abstract])) OR (Coronary Atheroscleroses[Title/Abstract])) OR (Coronary Atherosclerosis[Title/Abstract])) OR (Arteriosclerosis, Coronary[Title/Abstract]))) AND ((("Computed Tomography Angiography"[Mesh]) OR (((((((((Angiographies, Computed Tomography[Title/Abstract]) OR (Computed Tomography Angiographies[Title/Abstract])) OR (Tomography Angiographies, Computed[Title/Abstract])) OR (Tomography Angiography, Computed[Title/Abstract])) OR (Angiography, Computed Tomography[Title/Abstract])) OR (Angiography, CT[Title/Abstract])) OR (CT Angiography[Title/Abstract])) OR (Angiographies, CT[Title/Abstract])) OR (CT Angiographies[Title/Abstract]))))) AND (coronary) AND ("Coronary Angiography"[Mesh]) OR (((Angiography, Coronary[Title/Abstract]) OR (Angiographies, Coronary[Title/Abstract])) OR (Coronary Angiographies[Title/Abstract])) AND (invasive) Filters: Publication date from 2012/01/01 to 2022/05/01

**Supplementary Appendix 1:** Search terms for Embase.

| No. | Query | Results | Date |
| --- | --- | --- | --- |
| #15 | #3 AND #8 AND #13 AND #14 | 1190 | 1-May-22 |
| #14 | [2012-2022]/py | 16092512 | 1-May-22 |
| #13 | #11 AND #12 | 6556 | 1-May-22 |
| #12 | invasive | 720600 | 1-May-22 |
| #11 | #9 OR #10 | 55240 | 1-May-22 |
| #10 | 'artery disease, coronary':ab,ti OR 'artery diseases, coronary':ab,ti OR 'coronary artery diseases':ab,ti OR 'left main coronary artery disease':ab,ti OR 'left main disease':ab,ti OR 'left main diseases':ab,ti OR 'left main coronary disease':ab,ti OR 'coronary arteriosclerosis':ab,ti OR 'arterioscleroses, coronary':ab,ti OR 'coronary arterioscleroses':ab,ti OR 'atherosclerosis, coronary':ab,ti OR 'atheroscleroses, coronary':ab,ti OR 'coronary atheroscleroses':ab,ti OR 'coronary atherosclerosis':ab,ti OR 'arteriosclerosis, coronary':ab,ti | 19459 | 1-May-22 |
| #9 | 'coronary angiography'/exp | 37040 | 1-May-22 |
| #8 | #6 AND #7 | 21170 | 1-May-22 |
| #7 | coronary | 807871 | 1-May-22 |
| #6 | #4 OR #5 | 77174 | 1-May-22 |
| #5 | 'angiographies, computed tomography':ab,ti OR 'computed tomography angiographies':ab,ti OR 'tomography angiographies, computed':ab,ti OR 'tomography angiography, computed':ab,ti OR 'angiography, computed tomography':ab,ti OR 'angiography, ct':ab,ti OR 'ct angiography':ab,ti OR 'angiographies, ct':ab,ti OR 'ct angiographies':ab,ti | 22488 | 1-May-22 |
| #4 | 'computed tomographic angiography'/exp | 72703 | 1-May-22 |
| #3 | #1 OR #2 | 381092 | 1-May-22 |
| #2 | 'artery disease, coronary':ab,ti OR 'artery diseases, coronary':ab,ti OR 'coronary artery diseases':ab,ti OR 'left main coronary artery disease':ab,ti OR 'left main disease':ab,ti OR 'left main diseases':ab,ti OR 'left main coronary disease':ab,ti OR 'coronary arteriosclerosis':ab,ti OR 'arterioscleroses, coronary':ab,ti OR 'coronary arterioscleroses':ab,ti OR 'atherosclerosis, coronary':ab,ti OR 'atheroscleroses, coronary':ab,ti OR 'coronary atheroscleroses':ab,ti OR 'coronary atherosclerosis':ab,ti OR 'arteriosclerosis, coronary':ab,ti | 19459 | 1-May-22 |
| #1 | 'coronary artery disease'/exp | 377013 | 1-May-22 |

**Supplementary Table S1:** The Jadad Scale for assessing the quality for randomized controlled trials.

| Item/Study |  | Dewey2016 | Kofoed2021 | Maurovich2022 | Shen2020 |
| --- | --- | --- | --- | --- | --- |
| Randomization | Was the study described as randomized (this includes the use of words such as randomly, random, and randomization)? | ☆☆ | ☆ | ☆☆ | ☆☆ |
| Allocation concealment | Adequate concealment was that up to the point of treatment (eg, central randomization). | ☆ | ☆☆ | ☆☆ | ☆ |
| Double-blinding | Was the study described as double blind? | ☆ | ☆☆ | ☆☆ | ☆ |
| Dropouts and withdrawals | Defined, on the scale, as trial participants who were included in the study but did not complete the observation period or who were not included in the analysis (but should have been described). | ☆ |  |  | ☆ |
| Score |  | 5 | 5 | 6 | 5 |

**Supplementary Table S2:** Newcastle-Ottawa Scale for assessing the quality for cohort trials.

| Item/Study |  | Dewey2021 | Winther2022 |
| --- | --- | --- | --- |
| Selection | Representativeness of the exposed cohort | ☆ | ☆ |
|  | Selection of the non exposed cohort | ☆ | ☆ |
|  | Ascertainment of exposure | ☆ | ☆ |
|  | Demonstration that outcome of interest was not present at start of study | ☆ | ☆ |
| Comparability | Comparability of cohorts on the basis of the design or analysis | ☆ | ☆ |
| Exposure | Assessment of outcome |  | ☆ |
|  | Was follow-up long enough for outcomes to occur | ☆ | ☆ |
|  | Adequacy of follow up of cohorts | ☆ | ☆ |
| Score |  | 7 | 8 |

**Supplementary Table S3:** The scores of quality assessment for randomized controlled trials and cohort trials.

| Study | Study type | Sample | NOS | JADAD |
| --- | --- | --- | --- | --- |
| Dewey2016 | RCT | 329 |  | 5 |
| Dewey2021 | Cohort | 382 | 7 |  |
| Kofoed2021 | RCT | 584 |  | 5 |
| Maurovich2022 | RCT | 3561 |  | 6 |
| Shen2020 | RCT | 102 |  | 5 |
| Winther2022 | Cohort | 21590 | 8 |  |

NOS, Newcastle – Ottawa Scale; JADAD, Jadad Scale

**Supplementary Table S4:** Sensitivity analysis by detecting one study each time of Major adverse cardiac events.

|  | OR | 95%CI | *P* value | Heterogeneity |
| --- | --- | --- | --- | --- |
| Analysis after excluding Dewey 2016 performing via random-effects model | 1.40 | 1.07 to 1.83 | 0.01 | I2 =54%, P=0.07 |
| Analysis after excluding Dewey 2021 performing via random-effects model | 1.55 | 1.26 to 1.90 | ﹤0.0001 | I2 =22%, P=0.27 |
| Analysis after excluding Kofoed 2021 performing via random-effects model | 1.42 | 1.07 to 1.89 | 0.02 | I2 =47%, P=0.11 |
| Analysis after excluding Maurovich 2022 performing via random-effects model | 1.32 | 0.94 to 1.85 | 0.10 | I2 =58%, P=0.05 |
| Analysis after excluding Shen 2020 performing via random-effects model | 1.35 | 1.02 to 1.78 | 0.04 | I2 =60%, P=0.04 |
| Analysis after excluding Winther 2022 performing via random-effects model | 1.19 | 0.93 to 1.51 | 0.17 | I2 =0%, P=0.79 |

|  | OR | 95%CI | *P* value | Heterogeneity |
| --- | --- | --- | --- | --- |
| Analysis after excluding Dewey 2016 performing via random-effects model | 1.98 | 0.97 to 4.02 | 0.06 | I2 =61%, P=0.08 |
| Analysis after excluding Kofoed 2021 performing via random-effects model | 2.76 | 1.62 to 4.71 | 0.0002 | I2 =7%, P=0.34 |
| Analysis after excluding Maurovich 2022 performing via random-effects model | 1.60 | 1.05 to 2.46 | 0.03 | I2 =0%, P=0.46 |
| Analysis after excluding Shen 2020 performing via random-effects model | 2.31 | 1.19 to 4.48 | 0.01 | I2 =61%, P=0.08 |

**Supplementary Table S5:** Sensitivity analysis by detecting one study each time of Operation-related complications.

**Supplementary Figures**


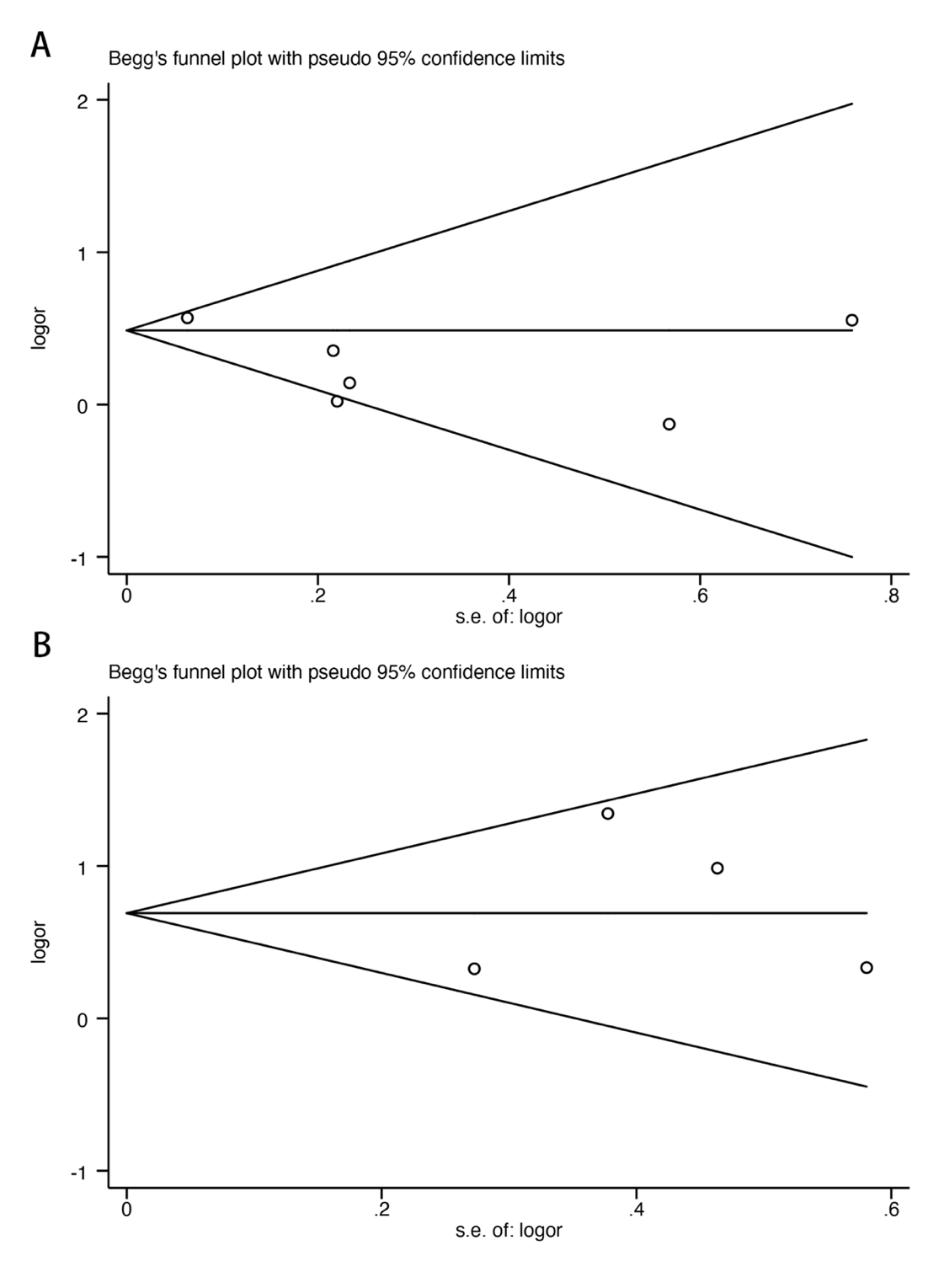
**Supplementary Figure S1:** Funnel plots for the assessment of publication bias in **(A)** Major adverse cardiac events, **(B)** Operation-related complications.
